# Supplementary material for: Multi‐dataset identification of innovative feature genes and molecular mechanisms in keratoconus
Source: J Cell Mol Med. 2024 Sep 19;28(18):e70079. doi: 10.1111/jcmm.70079 (PMC11412914; doi:10.1111/jcmm.70079)
Supplement: Supplementary file 2 — Material S2. [file JCMM-28-e70079-s002.zip › 7.GSEA analysis/3.DBNDD1/KEGG_INTESTINAL_IMMUNE_NETWORK_FOR_IGA_PRODUCTION.html]

Details for gene set KEGG\_INTESTINAL\_IMMUNE\_NETWORK\_FOR\_IGA\_PRODUCTION[GSEA]

|  || Dataset | DBNDD1.group.cls#h\_versus\_l |
| Phenotype | group.cls#h\_versus\_l |
| Upregulated in class | l |
| GeneSet | KEGG\_INTESTINAL\_IMMUNE\_NETWORK\_FOR\_IGA\_PRODUCTION |
| Enrichment Score (ES) | -0.50080824 |
| Normalized Enrichment Score (NES) | -1.329749 |
| Nominal p-value | 0.08863199 |
| FDR q-value | 0.78879637 |
| FWER p-Value | 0.949 |
Table: GSEA Results Summary

  

Fig 1: Enrichment plot: KEGG\_INTESTINAL\_IMMUNE\_NETWORK\_FOR\_IGA\_PRODUCTION      
 Profile of the Running ES Score & Positions of GeneSet Members on the Rank Ordered List

  

| SYMBOL | TITLE | RANK IN GENE LIST | RANK METRIC SCORE | RUNNING ES | CORE ENRICHMENT || 1 | CD86 | na | 1030 | 0.379 | 0.0148 | No |
| 2 | HLA-DRB5 | na | 1509 | 0.335 | 0.0404 | No |
| 3 | CCR10 | na | 4137 | 0.209 | -0.0108 | No |
| 4 | TNFRSF13C | na | 7128 | 0.122 | -0.0827 | No |
| 5 | ITGA4 | na | 7459 | 0.114 | -0.0788 | No |
| 6 | HLA-DMA | na | 8201 | 0.100 | -0.0885 | No |
| 7 | TNFRSF13B | na | 8984 | 0.086 | -0.1009 | No |
| 8 | ITGB7 | na | 9817 | 0.074 | -0.1162 | No |
| 9 | HLA-DRB4 | na | 10303 | 0.067 | -0.1223 | No |
| 10 | ICOSLG | na | 10476 | 0.065 | -0.1197 | No |
| 11 | HLA-DRB1 | na | 10490 | 0.065 | -0.1124 | No |
| 12 | IL15RA | na | 10985 | 0.058 | -0.1198 | No |
| 13 | TNFSF13B | na | 11445 | 0.053 | -0.1269 | No |
| 14 | HLA-DOA | na | 11835 | 0.049 | -0.1324 | No |
| 15 | TNFSF13 | na | 12671 | 0.041 | -0.1516 | No |
| 16 | HLA-DQB1 | na | 12864 | 0.039 | -0.1526 | No |
| 17 | LTBR | na | 14375 | 0.027 | -0.1929 | No |
| 18 | TNFRSF17 | na | 17975 | 0.003 | -0.2963 | No |
| 19 | AICDA | na | 19955 | -0.013 | -0.3519 | No |
| 20 | HLA-DOB | na | 21192 | -0.024 | -0.3847 | No |
| 21 | CD40 | na | 21562 | -0.028 | -0.3921 | No |
| 22 | CD28 | na | 21937 | -0.031 | -0.3992 | No |
| 23 | ICOS | na | 24698 | -0.066 | -0.4710 | No |
| 24 | IL6 | na | 24766 | -0.067 | -0.4650 | No |
| 25 | HLA-DRB3 | na | 25022 | -0.071 | -0.4640 | No |
| 26 | HLA-DRA | na | 26179 | -0.091 | -0.4867 | No |
| 27 | CXCL12 | na | 26671 | -0.101 | -0.4890 | Yes |
| 28 | HLA-DMB | na | 26948 | -0.107 | -0.4844 | Yes |
| 29 | CD80 | na | 27049 | -0.109 | -0.4745 | Yes |
| 30 | CCL28 | na | 27313 | -0.115 | -0.4686 | Yes |
| 31 | HLA-DPA1 | na | 28024 | -0.132 | -0.4736 | Yes |
| 32 | IL5 | na | 28795 | -0.151 | -0.4780 | Yes |
| 33 | CCR9 | na | 29179 | -0.162 | -0.4700 | Yes |
| 34 | IL2 | na | 29432 | -0.170 | -0.4573 | Yes |
| 35 | TGFB1 | na | 30914 | -0.218 | -0.4744 | Yes |
| 36 | IL15 | na | 31024 | -0.221 | -0.4516 | Yes |
| 37 | IL10 | na | 31214 | -0.229 | -0.4301 | Yes |
| 38 | CD40LG | na | 31492 | -0.240 | -0.4099 | Yes |
| 39 | CCL25 | na | 32045 | -0.266 | -0.3946 | Yes |
| 40 | MAP3K14 | na | 32153 | -0.273 | -0.3656 | Yes |
| 41 | HLA-DPB1 | na | 32889 | -0.320 | -0.3493 | Yes |
| 42 | PIGR | na | 32919 | -0.322 | -0.3123 | Yes |
| 43 | MADCAM1 | na | 33021 | -0.330 | -0.2764 | Yes |
| 44 | CXCR4 | na | 33022 | -0.330 | -0.2377 | Yes |
| 45 | IL4 | na | 33666 | -0.386 | -0.2108 | Yes |
| 46 | CCL27 | na | 33678 | -0.388 | -0.1655 | Yes |
| 47 | HLA-DQA2 | na | 34710 | -0.832 | -0.0975 | Yes |
| 48 | HLA-DQA1 | na | 34711 | -0.834 | 0.0005 | Yes |
Table: GSEA details [plain text format]

  

Fig 2: KEGG\_INTESTINAL\_IMMUNE\_NETWORK\_FOR\_IGA\_PRODUCTION      
 Blue-Pink O' Gram in the Space of the Analyzed GeneSet

  

Fig 3: KEGG\_INTESTINAL\_IMMUNE\_NETWORK\_FOR\_IGA\_PRODUCTION: Random ES distribution      
 Gene set null distribution of ES for **KEGG\_INTESTINAL\_IMMUNE\_NETWORK\_FOR\_IGA\_PRODUCTION**
